# Maximizing non-enzymatic methods for harvesting adipose-derived stem from lipoaspirate: technical considerations and clinical implications for regenerative surgery

**DOI:** 10.1038/s41598-017-10710-6

**Published:** 2017-08-30

**Authors:** Barbara Bellei, Emilia Migliano, Marinella Tedesco, Silvia Caputo, Mauro Picardo

**Affiliations:** 1 Laboratory of Cutaneous Physiopathology and Integrated Center of Metabolomics Research, San Gallicano Dermatologic Institute, IRCCS, Rome, Italy; 2 Department of Plastic and Reconstructive Surgery, San Gallicano Dermatologic Institute, IRCCS, Rome, Italy

## Abstract

In the past decade, adipose tissue has become a highly interesting source of adult stem cells for plastic surgery and regenerative medicine. The adipose source offers two options for the isolation of regenerative cells: the enzymatic digestion an expensive time-consuming procedure lacking a common standard operating protocol, or the non-enzymatic dissociation methods based on mechanical forces to break the processed adipose tissue. Here, we propose innovative inexpensive non-enzymatic protocols to collect and concentrate clinically useful regenerative cells from adipose tissue by centrifugation of the infranatant fraction of lipoaspirate as first step, usually discarded as a byproduct of the surgical procedure, and by fat shaking and wash as second enrichment step. The isolated cells were characterized according to the criteria proposed by the Mesenchymal and Tissue Stem Cell Committee of the International Society for Cellular Therapy (ISCT) to define human mesenchymal stem cells, and the results were compared with matched lipoaspirate samples processed with collagenase. The results demonstrated the usability of these new procedures as an alternative to fat grafting for treating stem cell-depleted tissues and for specific application requiring minimal or null soft tissue augmentation, such as skin diseases including severe burn and post-oncological scaring, chronic non-healing wounds, and vitiligo.

## Introduction

In the past years, aesthetic regenerative medicine has safely and effectively utilized authologous fat grafting to provide structural augmentation of the subcutaneous adipose layers and related tissues. Furthermore, studies on whole adipose tissue composed predominantly of mature adipocytes (90% of tissue volume and about two-thirds of the total cell number^1^), and a restricted portion of blood-derived cells, pericytes, smooth muscle cells and endothelial cells, have revealed the presence of pluripotent stem/progenitor cells, the so-called adipose-derived stem cells (ADSCs), capable of self-renewing and differentiating into a range of mesenchymal tissues^2, 3^. In addition, trans-differentiation of ADSCs into cells of non-mesenchymal origin, e.g. hepatocytes, neurons and pancreatic islet cells, has been observed *in vitro* when specific culture conditions and stimuli apply^4–8^. Human non-embryonic adult mesenchymal stem cells (MSCs), including blood, bone marrow and adipose-derived stem cells represent important cell resources and hold great promise for cell-based therapies, drug discovery, disease modeling, and pharmaceutical applications^9, 10^. However, higher mesenchymal stem cell concentration^11, 12^, ease and safely of access in the native adipose tissue complex, has lead most part of researchers and clinicians to transfer from the bone marrow sources to the adipose tissue. In addition, recent comparative analysis has demonstrated that ADSCs are more resistant to stress-induced senescence than bone marrow-derived stem cells and more effective in promoting neovascularization in animal models^13^. The greater therapeutic potential of the adipose tissue is also supported by the characterization of the adipose-derived stromal vascular fraction (AD-SVF), a source of ADSCs, endothelial progenitor cells, T cells, B cells, mast cells, and adipose-resident macrophages with repair and regenerative potential^14, 15^. So far, based on increasing understanding of the basic science of stem cells and encouraging experimental studies, the interest in non-manipulated (*in vitro*) fat grafting raised tremendously as well as the number of clinical applications have grown exponentially in the past 20 years. Authologous fat grafting has been successfully used for facial soft tissue deformity correction^16^, muscular regenerative therapies^17^, cosmetic and reconstructive breast surgery^18^, facial rejuvenation^19^, scleroderma^20^, post-oncological related defect remodeling^21, 22^, depressed scar problems and chronic ulcer^23^.

Most surgical protocols for fat grafting recommend a centrifugation step to remove excess fluids and unnecessary components, such as water, oil, and dead cells and anesthetic solution^24^. The relevance of graft tissue condensation seems to be related to volume retention and to the limitation of injected volume given that, excessive volume leads to severe ischemia and fat necrosis^12^. Moreover, because aspirated fat tissue is relatively poor in stem cells^14, 25^, condensation of ADSCs in the graft is crucial for the therapeutic outcome especially when the regenerative purpose (tissue repair) prevails in volumetric tissue restoration. An innovative method named cell-assisted lipotransfer (CAL), introduced by Matsumoto in 2006, proposes to increase the ADSCs/adipocyte ratio combining aspirated fat transplantation with enzymatically isolated ADSCs^26–28^. However, the use of enzymes such as collagenase, trypsin or dispase, is implies high costs and might impact on safety^29^ and efficacy^30^. Collagenase preparations have also been shown to activate human complement^31^, which could induce a local inflammatory reaction. Furthermore, enzymatic methods may cause stem cell differentiation^32^. So far, the translation of adipose stem cell-based therapies into clinical requires standard operating protocols for replacing the enzymes. Up to now in the clinical practice, three major non-enzymatic methods have been proposed to improve the ADSCs/adipocyte ratio: decantation (gravity sedimentation), centrifugation and filtration. Recently, mechanical disruption of tissue in a closed system to reduce the size of lipoaspirates^33, 34^, and lipoaspirate washing^35^ have been proposed alternative methods to avoid enzymatic digestion. However, both the proposed methods require large amounts of lipoaspirates, and even if these systems extract a consistent portion of ADSCs, up to now data report that the efficiency is significantly lower than enzymatic protocols and collagenase digestion is still considered the standard practice for research purposes. Moreover, the application of an external force, as proposed in the case of mechanical devices, could be very traumatic to cells reducing post-transplant engraftment. In the current study, we have evaluated new enzyme-free protocols to process small amount of lipoaspirates, resulting in an ADSCs-enriched product from both fluid and solid fractions of adipose tissue obtained by liposuction for innovative applications in regenerative medicine. Qualitative analysis demonstrated the usability of these new procedures as an alternative to fat grafting for treating stem cell-depleted tissues and for specific application requiring functional or structural tissue regeneration but minimal or null soft tissue augmentation such as skin diseases including chronic non-healing wounds, stable vitiligo, severe burn and post-oncological scaring.

## Results

### ADSCs quantification

**Figure 1:**
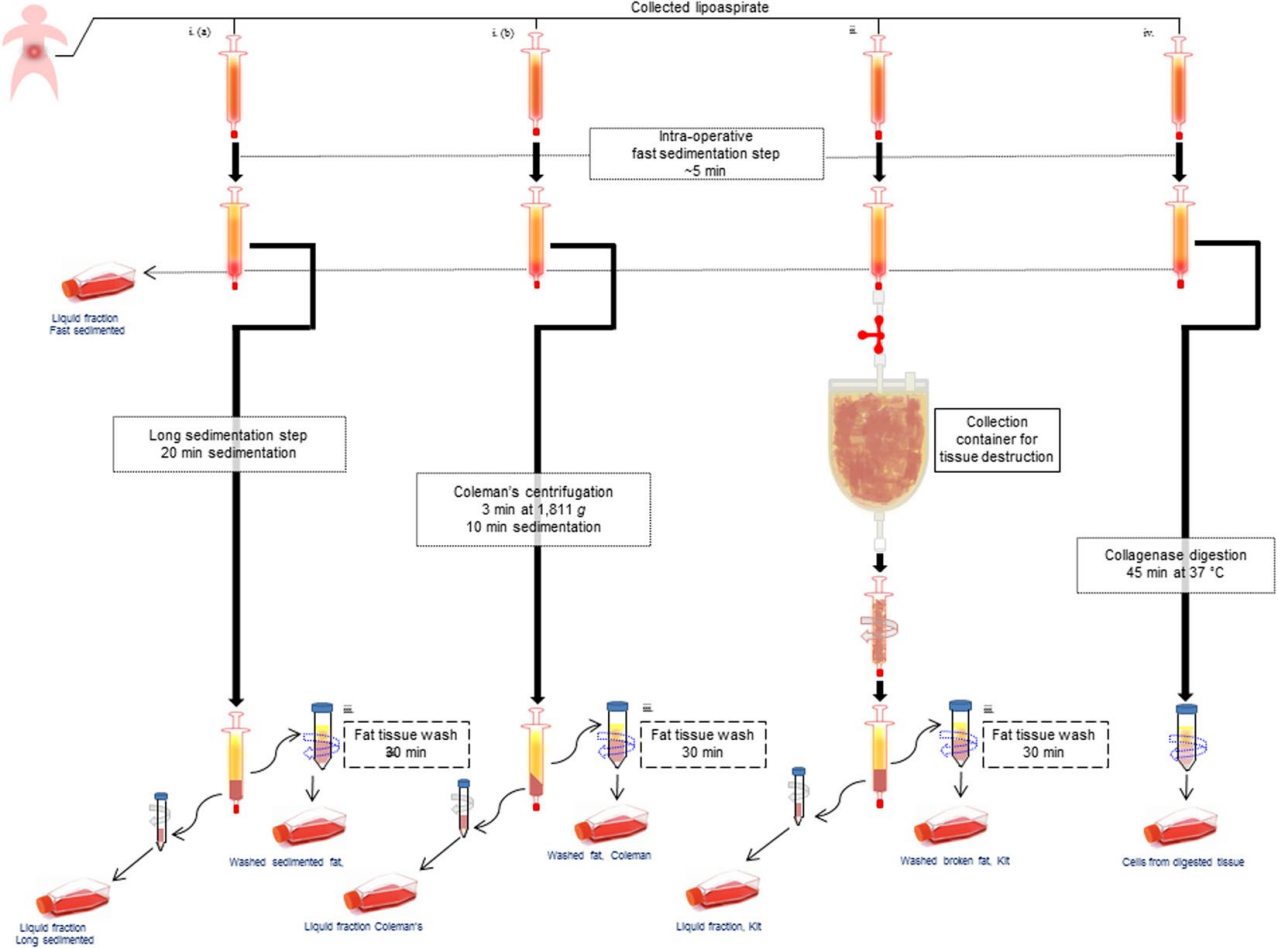
Schematic representation of methods used for ADSCs isolation. All samples were sedimentated for a short period before been processed with different protocols. i. (**a**) Sedimentation procedure to extract cells in the liquid fraction followed by fat wash. i. (**b**) Coleman’s methods was used to separate the liquid fraction and collect cells. Fat tissue was then washed. ii. The isolation kit a single use manual device, consisting of a tissue collection container for mechanical dissociation was used before recover the material in syringes. Broken fat was then washed to extract cells. iv. Collagenase digestion of lipoaspirates. As last step (omitted in the figure), before *in vitro* cell cultures, samples were treated with red blood cell lysis buffer and filtered through a 70 μm cell strainer and centrifuged. Finally, pellets were resuspended in culture medium. All the details are described in materials and methods section.

Cells isolated using different protocols (see details in Fig. 1 and material and methods section) were plated and cultured with 40% FBS in DMEM for 14 days before cell quantification. ADSCs were successfully obtained from all the specimens collected even if a huge individual variability was observed in terms of number of cells isolated per milliliter of liposuction aspirates (mean value 2.29 ± 2.80 × 10^5^ cells/mL; range 0.18–15.13 × 10^5^ cells/mL) (Table 1). At the time of count, cells displayed a mean viability of 91.8% ± 4.7% based on Trypan Blue exclusion assay. To find out whether there is a correlation between age (range 16–74 years; mean age 50.2) and cell yield we performed a Pearson correlation analysis which indicates that there is no significant correlation between age and the general output of cell cultures measured as the mean number of viable cells per milliliter of liposuction collected considering all the isolation methods tested (correlation coefficient of −0.001) or as mean number of cells obtained by adipose tissue wash procedure (correlation coefficient of 0.028), or as mean number of cells obtained from the aqueous phase by centrifugation (correlation coefficient of −0.105). Comparative analysis of different isolation systems shows that the centrifugation step according to Coleman’s technique^36^ did not significantly modify the quantity of cells collected from lipoaspirates, since combining cells extracted from dry tissue wash with cells collected from the liquid fraction, we obtained a mean number of 5.11 ± 6.02 × 10^5^ cells/mL whereas, 5.13 ± 7.09 × 10^5^ cells/mL were isolated with the same procedures after a simple sedimentation step of harvested samples (p = 0.95). Similarly, the independent analysis of data regarding dry fat tissue and the liquid portion of lipoaspirates revealed that the quantity of cells achieved by fat wash after Coleman’s centrifugation was comparable to the number of cells isolated by fat wash of sedimented lipoaspirate (3.35 ± 4.76 × 10^5^ cells/mL and 3.98 ± 7.07 × 10^5^ cells/mL respectively) (see Table 1 for details). According to quantitative results, after the centrifugation with Coleman’s method or after the sedimentation step samples appeared similar and aqueous and solid fractions were equally distributed (Suppl. Figure 1). The liquid discarded during harvesting time of the surgical procedure, after few minutes of sedimentation, contains on average a lower number of cells (0.31 ± 0.31 × 10^5^ cells/mL) in comparison to all the other fractions. Overall the data demonstrated that standard surgical liposuction procedure drains away in the liquid fraction a significant number of stem cells (mean value of Coleman’s centrifugation/long sedimentation/fast sedimentation-derived fluid partitions 1.92 ± 1.78 × 10^5^ cells/mL), corresponding to 18.7% of the total number of cells isolated in absence of enzymatic digestion normalized as mL of lipoaspirate. As shown, this component of ADSCs could be easily enriched by a procedure of fat tissue wash that collect cells resident in the adipose stroma but probably not strictly associated to the tissue. In a subset of donors (17 out of 33 donors, mean age 48.0 years and presenting a mean value 2.33 ± 2.06 × 10^5^ cells/mL collected; range 0.17–7.52 × 10^5^ cells/mL) we also tested a commercially available single-use clinical-grade device for cell isolation from adipose tissue by mechanical disruption and filtration (Fastem, Corios) and we compared the results with the home-made methods used. In this group, we observed a moderate but not significant increase in the number of cells released in the fluid portions of liposuction aspirates (3.04 ± 4.43 × 10^5^ cells/mL kit method; 1.85 ± 1.83 × 10^5^ cells/mL p = 0.275 Coleman’s method; and 0.87 ± 0.90 × 10^5^ cells/mL p = 0.103 sedimentation method). By contrast, the number of cells extracted by fat wash was not modified (2.43 ± 2.43 × 10^5^ cells/mL kit; 2.83 ± 3.47 × 10^5^ cells/mL p = 0.690 Coleman’s method; and 3.87 ± 4.78 × 10^5^ cells/mL p = 0.283 sedimentation method) suggesting that the surplus cells released by the rupture of the tissue was entirely collected in the fluid part of lipoaspirates.

**Table 1:** Quantitative analysis of cells isolated with different harvest techniques

Patient	Age/Sex	Mean 10^5^ cells/mL	Liquid Fraction Fast Sedimented (n = 33) 10^5^ cells/mL	Liquid Fraction Coleman (n = 33) 10^5^ cells/mL	Wash fat Coleman (n = 33) 10^5^ cells/mL	Liquid Fraction Long Sedimented (n = 33) 10^5^ cells/mL	Wash fat Sedimented (n = 33) 10^5^ cells/mL	Liquid Fraction Kit (n = 17) 10^5^ cells/mL	Wash fat Kit (n = 17) 10^5^ cells/mL
L1	46/F	15.13 ± 15.87	0.06	7.80	25.0	4.80	38.0		
L2	63/F	1.27 ± 2.53	0.005	0.14	5.80	0.09	0.32		
M3	54/F	1.17 ± 0.91	0.44	0.58	1.75	0.58	2.50		
L4	74/F	0.46 ± 0.24	0.25	0.47	0.27	0.47	0.86		
L5	54/F	0.90 ± 0.71	0.13	1.40	1.69	0.18	1.10		
L6	50/M	1.75 ± 2.30	0.35	0.005	3.40	0.005	4.97		
L7	56/F	1.18 ± 1.31	0.005	0.37	3.10	0.46	1.97		
L8	61/F	1.36 ± 1.79	0.32	0.10	4.09	0	2.30		
L9	46/F	0.99 ± 0.62	0.23	0.94	1.63	0.55	1.61		
L10	54/F	1.37 ± 1.42	0.38	0.29	3.50	0.51	2.20		
L11	59/F	0.81 ± 0.48	0.17	0.59	1.20	0.71	1.37		
L12	26/M	0.72 ± 1.38	0.003	3.19	0.05	0.31	0.05		
L13	62/F	1.81 ± 1.83	0.55	1.50	4.68	0	2.35		
L14	47/F	3.31 ± 2.80	0.12	3.67	2.06	7.81	2.70		
L15	49/F	2.03 ± 1.40	0.13	1.18	3.74	2.33	2.78		
L16	41/F	1.89 ± 1.19	0.10	4.34	0.41	4.24	0.38		
LL1	26/M	1.01 ± 0.96	0.26	0.34	1.80	0.18	2.80	0.90	0.80
LL2	49/M	0.54 ± 0.63	0.19	1.83	0.53	0.001	0.47	0.001	0.75
LL3	16/M	0.18 ± 0.12	0.001	0.18	0.30	0.05	0.32	0.18	0.27
LL4	36/F	7.34 ± 10.71	1.65	5.14	1.70	0.05	7.00	17.4	4.90
LL5	56/M	1.86 ± 1.20	0.46	2.07	2.90	1.17	3.20	1.70	0.38
LL6	61/M	1.35 ± 0.79	0.30	1.67	1.65	2.00	0.15	2.12	1.60
LL7	56/M	3.53 ± 3.57	0.28	1.16	4.05	0.70	4.30	10.7	3.50
LL8	59/F	6.21 ± 7.05	0.23	6.3	7.90	2.53	20.9	7.90	6.30
LL9	48/M	5.1 ± 5.25	0.45	2.85	14.8	2.34	4.20	14.8	2.85
LL10	47/M	2.42 ± 2.58	0.66	0.39	4.17	0.005	6.83	4.17	0.39
LL11	46/M	1.43 ± 0.92	0.005	1.30	0.36	1.00	1.10	1.70	1.36
LL12	56/F	1.49 ± 1.06	0.55	1.30	1.70	1.00	3.24	0.36	1.30
LL13	30/F	0.73 ± 0.51	0.76	0.61	0.76	0.54	1.80	0.76	0.61
LL14	63/F	0.5 ± 0.39	0.60	0.38	0.12	0.32	0.73	0.12	0.38
LL15	59/F	1.58 ± 1.50	0.22	0.78	1.33	2.40	4.60	1.33	0.78
LL16	53/M	2.35 ± 2.07	0.10	0.66	2.55	0.24	2.30	2.55	0.66
LL17	61/F	1.92 ± 1.57	0.30	4.57	1.45	0.32	1.92	1.45	4.57
Mean value ± SD	52	2.29 ± 2.80	0.31 ± 0.31	1.76 ± 1.93	3.35 ± 4.76	1.15 ± 1.68	3.98 ± 7.07	3.04 ± 4.43	2.43 ± 2.43

Comparative analysis of cell yield after 14 days of *in vitro* cell culture. Data presented results from single donors and median ± SD for each separation protocol. Cell yields were normalized by dividing the cell number by the initial volume (in mL) of the lipoaspirate portion. n = number of patients analyzed.

### Phenotypic characterization by flow cytometry

**Figure 2:**
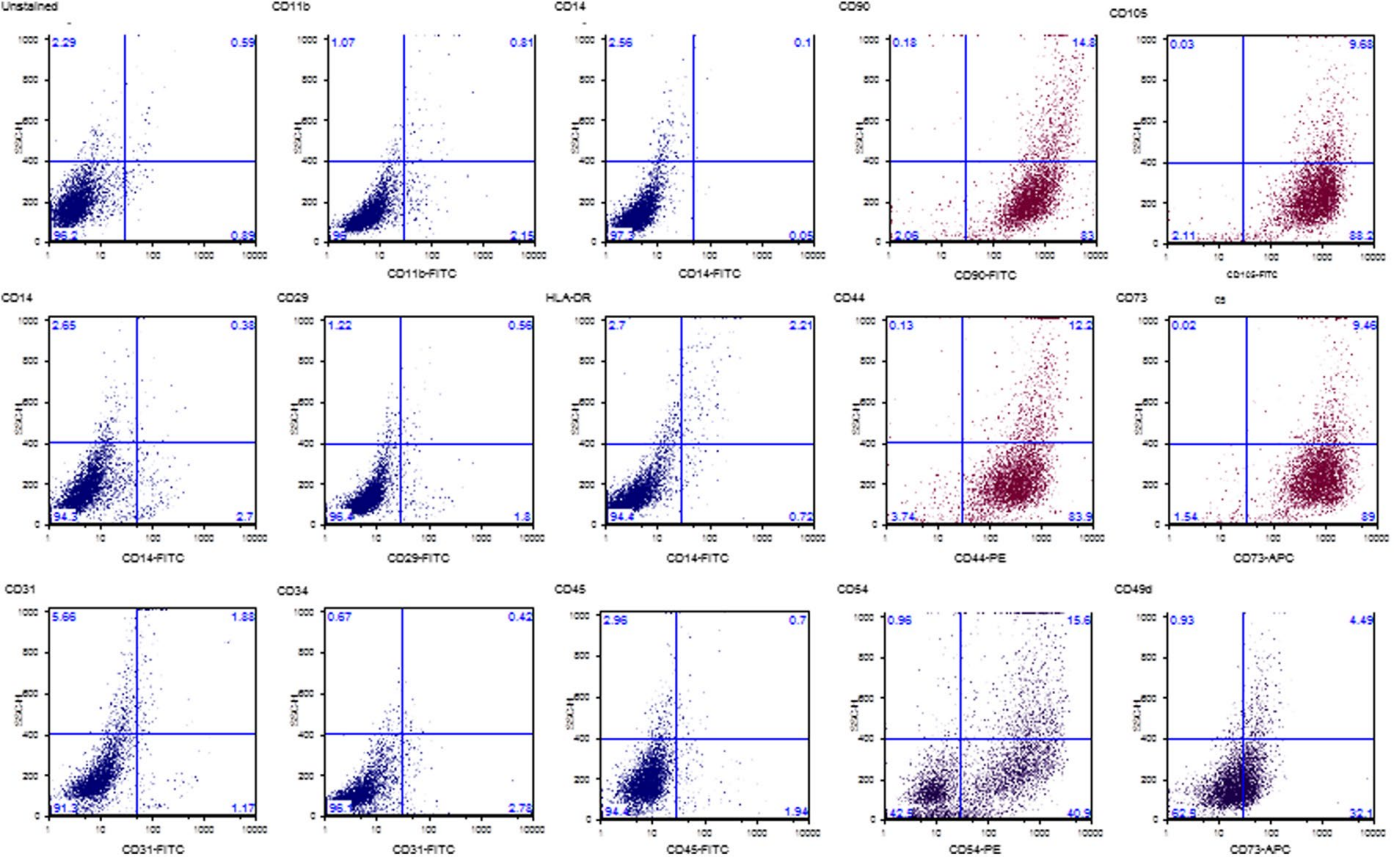
Expression of surface markers detected by flow cytometric analysis of cultured ADSCs. Primary cultures of ADSCs were expanded to passage 2 in DMEM 20% FBS and analyzed before reaching the confluence. The percentage of positive cells for each marker was calculated after subtraction of the non-specific fluorescence obtained with the isotype control antibodies. Data shows a representative set of dot-plot from one individual in each isolation protocol.

Next, we analyzed a set of 13 surface markers including those described by the Mesenchymal and Tissue Stem Cell Committee of the International Society for Cellular Therapy (ISCT) as specific immunonological characterization of multipotent mesenchymal stromal cells^37, 38^. Culture-expanded ADSCs from each group of isolation methods expressed comparable levels (greater than 95%) of CD44, CD105, CD73, CD90 mesenchymal markers and were negative (≤3%) for the hematopoietic markers CD45, CD19, CD34, CD31, CD14, CD11b and HLA-DR (Table 2). The expression of CD73 and CD105 also excluded the contamination of cell cultures with preadipocytes since these surface markers are not expressed by committed preadipocytes and mature adipocytes^39^. In addition, we investigated the expression of CD49d (integrin α4) and of CD54 (ICAM-I), two adhesion molecules previously found to be highly expressed in adipose-derived stem cells and minimally expressed in bone marrow-derived stem cells^3, 40^. Both surface markers were found on cells of all isolation groups even if a donor heterogeneity was observed. Representative single cell culture FACS data for staining intensities are shown in Fig. 2.

**Table 2:** Immunophenotypic characterization of ADSCs. Data are representative of analysis of eleven individuals.

Antigen	Liquid Fraction Fast sedimented (n = 33)	Liquid Fraction Coleman (n = 33)	Liquid Fraction Long Sedimented (n = 33)	Liquid Fraction Kit (n = 17)	Wash fat Coleman (n = 33)	Wash fat Sedimented (n = 33)	Wash fat Kit (n = 17)	Collagenase (n = 10)
CD105	98% ± 4	98% ± 3	97% ± 6	98% ± 2	99% ± 6	98% ± 5	97% ± 4	98% ± 4
CD90	99% ± 1	99% ± 1	99% ± 1	98% ± 2	100% ± 0	99% ± 1	98% ± 1	97% ± 2
CD73	99% ± 2	100% ± 1	99% ± 3	99% ± 1	98% ± 0	99% ± 1	98% ± 1	98% ± 1
CD44	97% ± 1	99% ± 1	99% ± 1	99% ± 1	100% ± 0	99% ± 1	98% ± 1	99% ± 1
CD54	56% ± 9	59% ± 6	54% ± 12	73% ± 14	49% ± 11	51% ± 20	59% ± 16	66% ± 17
CD49d	17% ± 5	22% ± 8	24% ± 4	27% ± 11	31% ± 9	26% ± 6	11% ± 13	27% ± 8
CD45	2% ± 1	3% ± 2	3% ± 1	2% ± 2	3% ± 1	3% ± 1	1% ± 2	2% ± 2
CD34	2% ± 1	1% ± 1	0% ± 0	0% ± 0	0% ± 0	1% ± 1	0% ± 0	0% ± 0
CD31	1% ± 0	1% ± 0	1% ± 0	1% ± 0	2% ± 0	1% ± 1	1% ± 0	3% ± 2
CD14	1% ± 1	2% ± 1	0% ± 0	1% ± 0	0% ± 0	0% ± 0	0% ± 0	0% ± 0
CD11a	0% ± 0	1% ± 0	1% ± 0	0% ± 0	1% ± 1	0% ± 0	0% ± 0	1% ± 1
HLA-DR	0% ± 0	0% ± 0	0% ± 0	0% ± 0	1% ± 0	0% ± 0	0% ± 0	0% ± 0
CD29	2% ± 3	3% ± 5	1% ± 2	3% ± 3	3% ± 3	3% ± 3	2% ± 1	2% ± 2

Flow cytometric analysis of ADSCs for positive (CD105, CD90, CD73 and CD44) and negative (CD45, CD34, CD14, CD11a, HLA-DR and CD29) surface markers. The expression of two adhesion molecules previously found to be highly expressed in adipose-derived stem cells (CD49d and CD54) and minimally expressed in bone marrow-derived stem cells was also evaluated.

### ADSCs rate of cell proliferation

The effect of procedures used for cell isolation on growth characteristics was evaluated. In general, under the cell culture conditions used in this study, long term observation showed an exponential growth phase (between passages 1 and 10) after which cell proliferation progressively declined (data not shown). So far, cell cultures obtained with different methods from the same donor (n = 6) at passages 2 and 6, including sample derived from collagenase digestion as control, were seeded at the density of 2 × 10^4^ cells/cm^2^ and cultured in DMEM containing 20% FBS for 3 and 5 days before cell count and Trypan Blu exclusion assay. In all cases, including samples treated with the isolation system using physical force, there was no difference in the proliferation capacity and cell vitality (Table 3).

**Table 3:** Analysis of the proliferation rate of ADSCs. These data are representative of six individuals

Passage#2% Increased number of cells over T0	3 days	5 days
Fast Sed Liquid	165 ± 18	266 ± 22
Liquid Fraction Coleman	183 ± 21	241 ± 31
Liquid Fraction Sedimented	211 ± 17	248 ± 25
Wash Fat Coleman	195 ± 16	255 ± 21
Wash Fat Sedimented	201 ± 27	267 ± 29
Collagenase	189 ± 38	237 ± 22
Passage#6% Increased number of cells over T0	3 days	5 days
Fast Sed Liquid	147 ± 19	251 ± 28
Liquid Fraction Coleman	177 ± 9	244 ± 19
Liquid Fraction Sedimented	195 ± 20	256 ± 30
Wash Fat Coleman	199 ± 12	216 ± 25
Wash Fat Sedimented	218 ± 23	233 ± 16
Collagenase	182 ± 19	249 ± 8

### Anchorage-independent spheres formation

**Figure 3:**
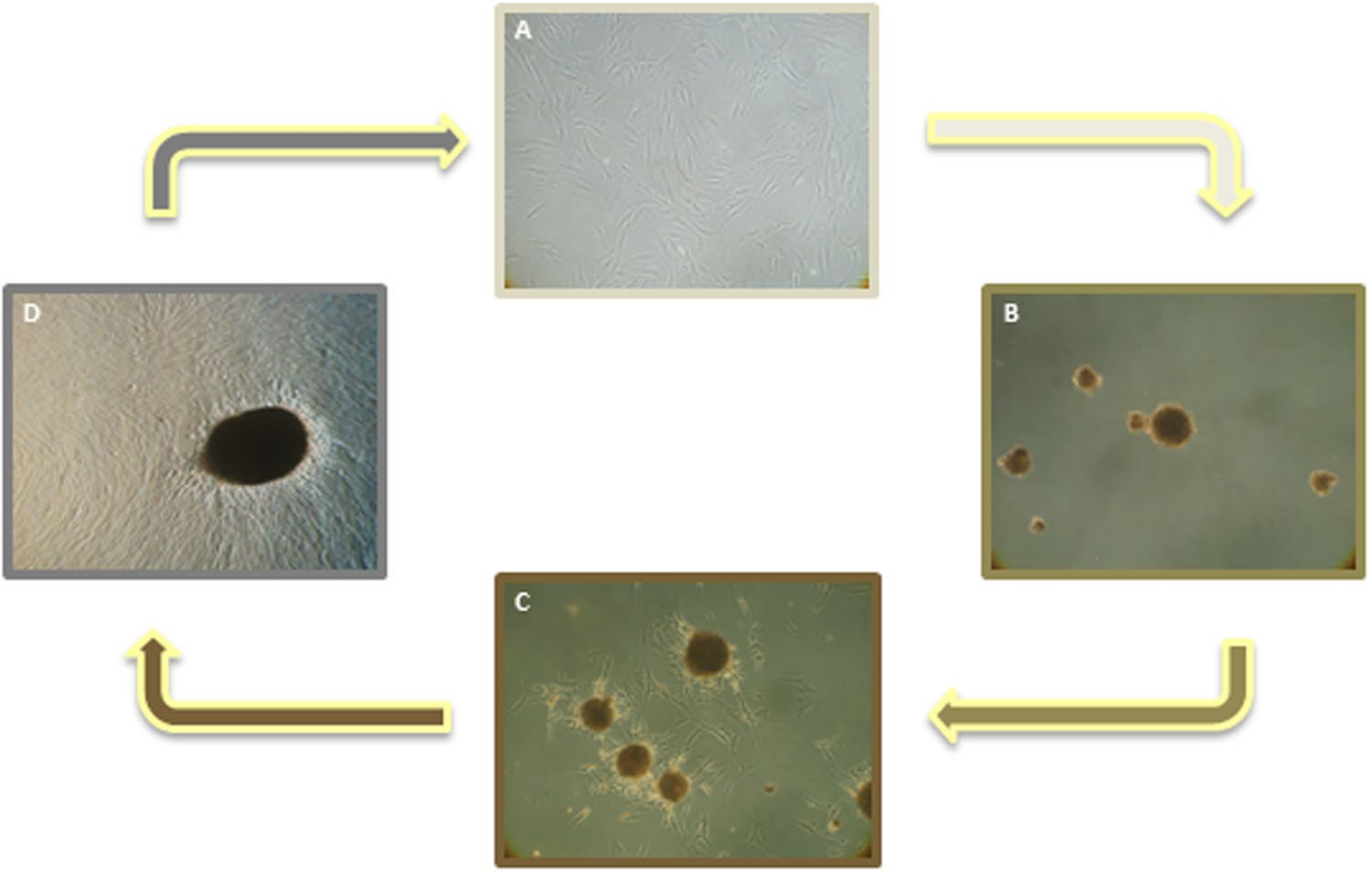
Development of anchorage-independent multicellular spheroids and induction of reversal into monolayer. (**A**) Microscopic images of ADSCs as monolayer. (**B**) Clusters of ADSCs grow in suspension as anchorage-independent multicellular spheroids at day 7. (**C**) Induction of reversal of spheroids into monolayer (2 days). (**D**) Progressive reversal of a spheroid over 1 week to reach complete reversal. Original magnification 20x.

The cells were detached by trypsin digestion and resuspended in DMEM containing 40% FBS and transfer to untreated tissue culture plate. Within 2 days the cells, kept in a low-adhesion culture condition, gave rise to floating spherical cell aggregates (considered a hallmark of the stemness feature^41^) with a well-delineated border (Fig. 3). Increasing evidence suggest that the 3D culture system recapitulates the tissue microenvironment promoting intercellular organization and function specific to stem cells aggregates since a similar spontaneous process of 3D sphere assembly has been observed *in vivo* when human mesenchymal stem cells (MSCs) are injected into the peritoneum of mice^42, 43^. Under these condition, the rate of cell proliferation is extremely low (data not shown) compared to the cells growth in adhesion and cells progressively gain a quiescent-like state resembling the physiological dormant state described within the cellular niche of adult stem cell lineages^44^. When transferred back to adhesion conditions the cells spread out and a monolayer culture of fibroblast-like cells formed maintaining the surface expression profile and the multilineage differentiation potential (data not shown).

### *In vitro* differentiation of ADSCs

**Figure 4:**
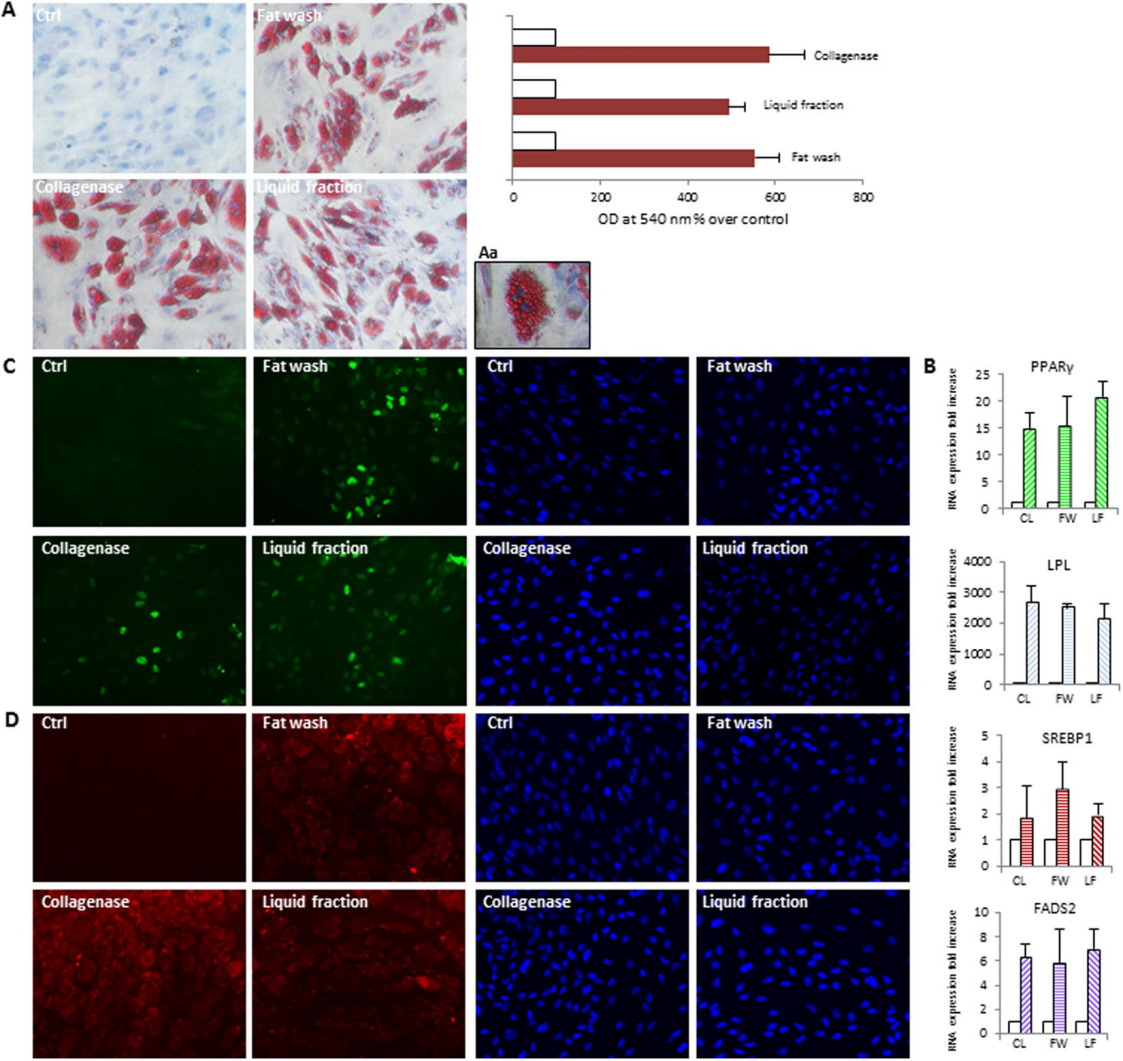
Analysis of adipogenically induced ADSCs isolated from the liquid fraction, the washed fat, or collagenase digested tissue as control. Since no difference was observed comparing cells obtained after the sedimentation or the centrifugation (Coleman’s method) as a first step, data were combined for quantitative analysis. Similarly, images are representative of each category of isolation protocols. (**A**) Oil Red staining. Light microscopy images were captured before semi-quantification. (Aa) enlarged view showing intracellular neutral lipid accumulation in detail. The stains from both uninduced and induced cultures were extracted using isopropanol and quantified by spectrophotometry. Results are reported as x-fold increase compared with undifferentiated control cells. Graphs represent mean values from eight individual donors, assayed in duplicate. Error bars represent SD from eight individual donors. (**B**) Semi-quantitative RT-PCR analysis for expression of adipogenic genes peroxisomal proliferator-activated receptor (PPARγ), lipo protein lipase (LPL), sterol regulatory element-binding protein 1 (SREBP1), and fatty acid desaturase 2 (FADS2). Glyceraldehyde-3-phosphate dehydrogenase (GAPDH) expression was used to normalize cDNA concentration for each sample set. Graphs and SD are from sixteen patients analyzed. The expression of PPARγ (**C**) and SREBP1 (**D**) were additionally confirmed at protein level by immunofluorescence analysis in several independent experiments. Nuclei were labelled with bisbenzidine (DAPI). Original magnification 20x. Ctrl, undifferentiated control; CL, collagenase; FW, fat wash; LF, liquid fraction.

**Figure 5:**
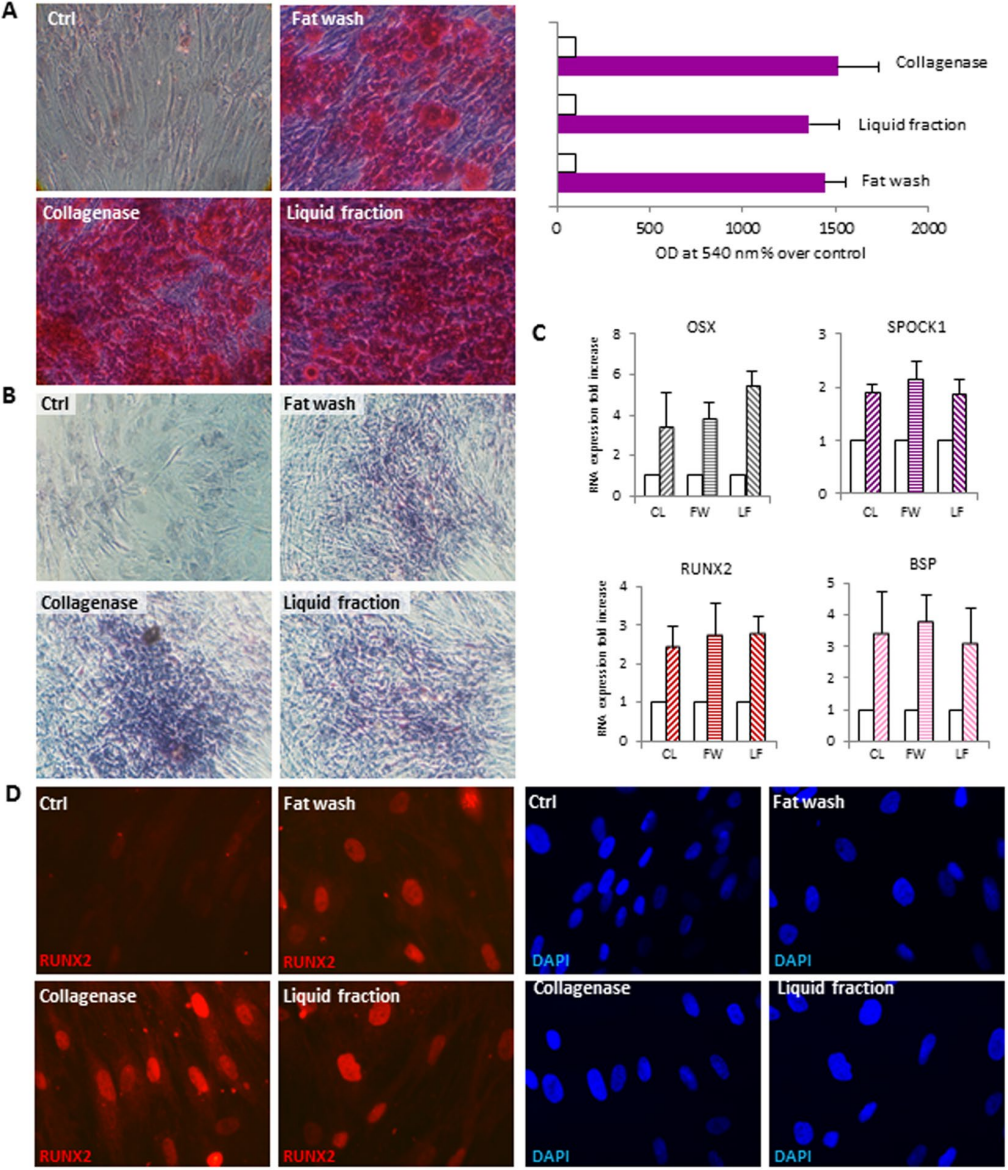
Analysis of osteogenically induced ADSCs isolated from the liquid fraction, the washed fat, or collagenase digested tissue as control. Since no difference was observed comparing cells obtained after the sedimentation or the centrifugation (Coleman’s method) as a first step, data were combined for quantitative analysis. Similarly, images are representative of each category of isolation protocols. (**A**) Alizarin red S staining. Light microscopy images were captured before semi-quantification. The stain from both uninduced and induced cultures were extracted using acetic acid and ammonium hydroxide and colometrically estimated measuring absorbance at 540 nm. Graphs represent mean values from eight individual donors, assayed in duplicate. Error bars represent SD from eight individual donors. (**B**) Alkaline phosphatase (AP) activity staining indicated a successful differentiation of ADSCs. Undifferentiated confluent ADSCs are slightly violet indicating a weak AP activity. (**C**) Semi-quantitative RT-PCR analysis for expression of osteogenic specific bone sialophosphoprotein (BSP), runt-related transcription factor (RUNX) 2, cwcv and kazal-like domains proteoglycan (SPOCK1), osterix (Osx). Glyceraldehyde-3-phosphate dehydrogenase (GAPDH) expression was used to normalize cDNA concentration for each sample set. Graphs and SD are from sixteen patients analyzed. The expression of most specific osteogenic transcription RUNX2 (**D**) was confirmed at protein level by immunofluorescence analysis in several independent experiments. Nuclei were labelled with bisbenzidine (DAPI). Original magnification 20X (**A**,**B**) and 40x (**C**). Ctrl, undifferentiated control; CL, collagenase; FW, fat wash; LF, liquid fraction.

**Figure 6:**
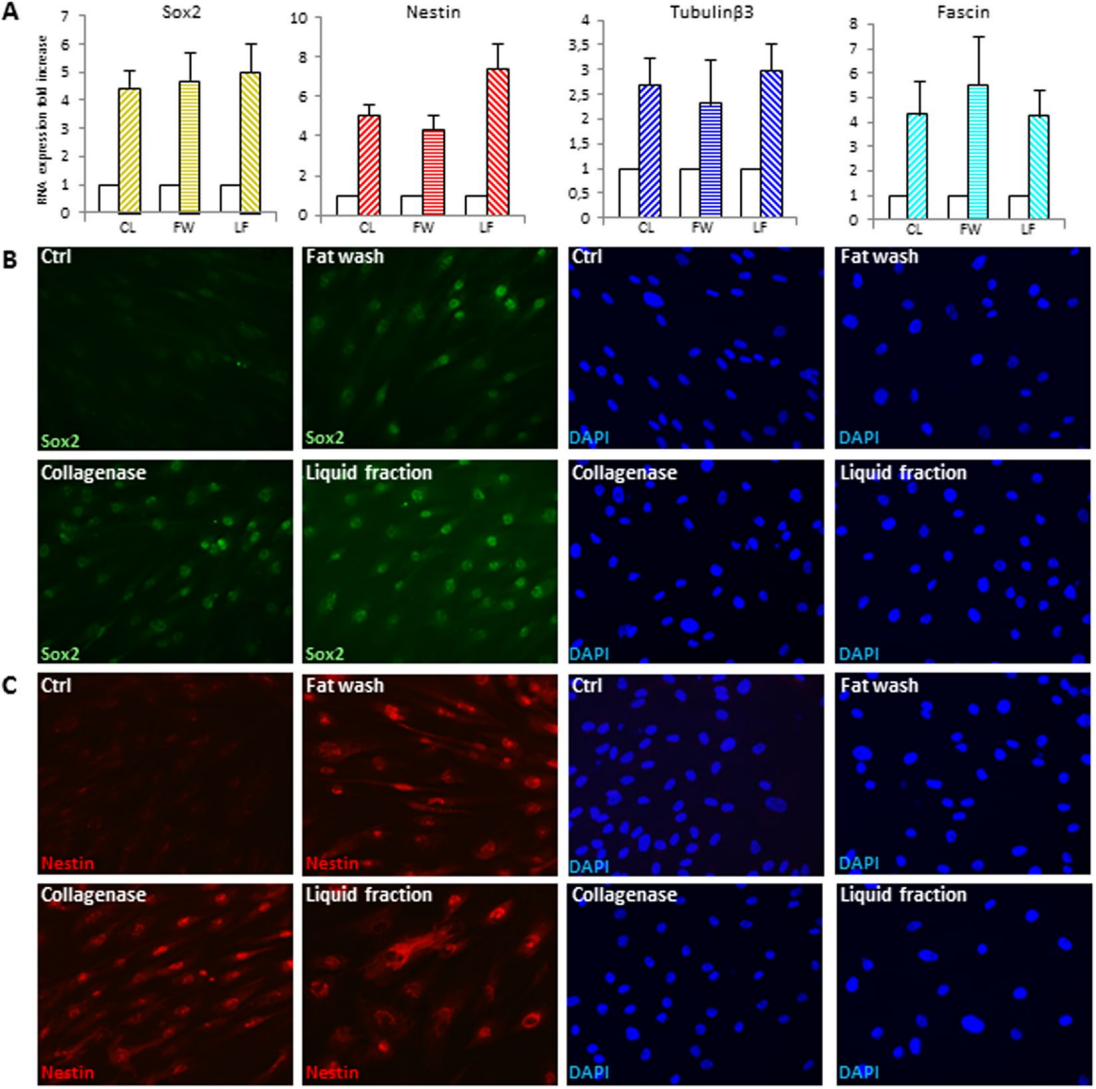
Differentiation of ADSCs into cells of neuronal lineage. (**A**) Semi-quantitative RT-PCR analysis for the expression of neurogenic lineage-specific genes sex determining region Y-box 2 (Sox-2), fascin, nestin, tubulinβIII. Glyceraldehyde-3-phosphate dehydrogenase (GAPDH) expression was used to normalize cDNA concentration for each sample set. Graphs and SD are from nine patients analyzed. The expression of Sox2 (**B**) and nestin (**C**) was also verified by immunofluorescence analysis in several independent experiments. Nuclei were labelled with bisbenzidine (DAPI). Original magnification 40x. Ctrl, undifferentiated control; CL, collagenase; FW, fat wash; LF, liquid fraction.

**Figure 7:**
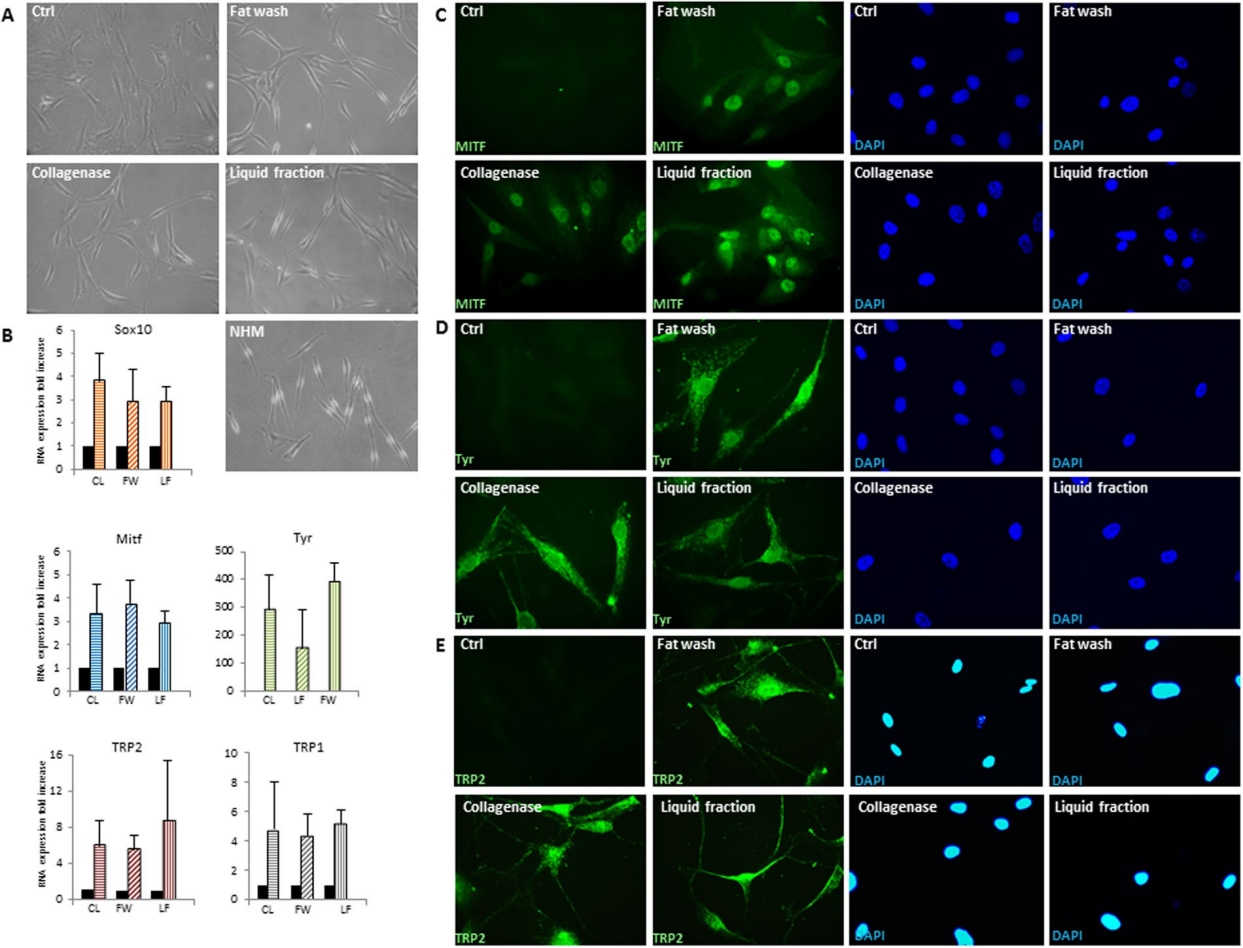
Analysis of ADSCs isolated from the liquid fraction, the washed fat, or collagenase digested tissue as control and cultured in medium for human epidermal melanocytes. (**A**) Microscopic images of ADSCs in DMEM 10% FBS and in melanocyte growth medium showing morphological modification after 2 days. Microscopic image of normal human melanocytes (NHM) is also provided. (**B**) Semi-quantitative RT-PCR analysis for expression of melanocyte specific genes Microphthalmia-associated protein (MITF), tyrosinase (Tyr), and tyrosinase-related protein 2 (TRP2). Glyceraldehyde-3-phosphate dehydrogenase (GAPDH) expression was used to normalize cDNA concentration for each sample set. Graphs and SD are from nine patients analyzed. The expression of MITF (**C**), Tyr (**D**) and TRP2 (**E**) was additionally confirmed at protein level by immunofluorescence analysis in several independent experiments. Nuclei were labelled with bisbenzidine (DAPI). Original magnification 20X (**A**) and 40x (**C**–**D**). Ctrl, undifferentiated control; CL, collagenase; FW, fat wash; LF, liquid fraction.

To compare the multipotency of ADSCs isolated from tissue and aqueous fractions using different methods, we tested their capacity for mesenchymal differentiation using standard *in vitro* tissue culture-differentiating conditions. At confluence, induction of adipocyte differentiation by appropriate treatment led to conversion of stem cells, morphologically similar to fibroblasts, to a spherical shape accumulating lipid droplets and to the acquisition of biochemical characteristics of the mature white adipocyte. The amount of lipid synthesis and quantitative expression analysis of the most specific adipogenic transcription factor peroxisome proliferator-activated receptor γ (PPARγ), of sterol regulatory element-binding protein 1 (SREBP1), lipo protein lipase (LPL) and fatty acid desaturase 2 (FADS2), demonstrated a similar differentiation capacity of all cell populations (Fig. 4). Similarly, under osteogenic conditions all stem cell lines differentiated successfully onto osteogenic lineage expressing genes and proteins associated with the osteoblast phenotype such as the key osteogenic transcription factor Runt-related transcription factor 2 (Runx-2), osterix (Osx), bone sialoprotein (BSP), sparc/osteonectin, cwcv and kazal-like domains proteoglycan (testican) 1 (SPOCK1), alkaline phosphatase (AP) and depositing a hydroxyapatite-mineralized extracellular matrix (Fig. 5). In addition, we investigated the capacity of these ADSCs to be committed into non-mesenchymal origin cell types. Neuronal induction of ADSCs resulted in the transition of cells into a neuronal morphology and expression of early markers of neuronal lineage including Sox2, nestin and tubulin βIII (Fig. 6) in the absence of significant differences among cells isolated by different protocols. In the presence of growth factors promoting melanocytic differentiation, ADSCs immediately acquired an elongated morphology and dendritic shape similar to fully differentiated adult melanocytes, whereas the expression of transcripts for proteins involved in normal pigmentation (Mitf/microftalmia transcription factor and sox10/) or specific to melanosomes (Tyr/tyrosinase, Tyrp1/tyrosinase-related protein 1 and Tyrp2/tyrosinase-related protein 2), were detected after five/six weeks at mRNA level and after eight weeks at protein level (Fig. 7). The global expression profiling confirmed that all stem cell lines were successfully committed to the melanocyte lineage.

## Discussion

The original concept of adipose tissue transplant as filler has evolved in recent years and it is now considered an important source of MSCs. The versatile differentiation potential, coupled with their abundance, increased enormously the possible applications of ADSCs especially in the research area of skin regeneration. In this particular field of regenerative medicine, requiring minimal or null soft tissue augmentation but significant functional or structural tissue repair, there is an urgent need to define innovative technologies to concentrate adult stem cells for authologous graft in clinical use. This is the case in chronic non-healing wounds, stable vitiligo, severe burn and post-oncological scaring. In this study, we evaluated the quantity and the quality of ADSCs collected by liposuction followed by laboratory-assisted cell enriched protocols, also considering the liquid fraction of lipoaspirate, currently discarded as a by-product in the clinical practice. We demonstrated that a significant number of fat-free ADSCs, presenting surface markers and biological characteristics similar to ADSCs isolated by enzymatic digestion, could be easily recovered from fluid portion of lipoaspirates and/or by fat tissue wash. The presence in the liquid fraction of processed lipoaspirate of a cell population exhibiting similar phenotypic properties to ADSCs harvested with collagenase has been previously reported^45, 46^. However, since this initial description, few groups have employed non-enzymatic methods to isolate and study ADSCs from lipoaspirates. One possible explanation for the presence of a pool of stem cells released in the fluid portion of the adipose complex is the mechanical injury occurring during the liposuction procedure and the action of endogenous enzymes. According to the idea that a substantial portion of ADSCs is released into the fluid portion of liposuction aspirates Matsumoto and co-worker (2006), reported that aspirated fat tissue contains only one-half the number of ADSCs compared to intact fat tissue^26^. The cellular abundance of the blood/saline portion of lipoaspirate waste is also explained by the perivascular origin of ADSCs^47^. However, we could not exclude the hypothesis that physiologically a portion of mesenchymal stem cells resides in the adipose tissue in a very dynamic fashion, in absence of a tight association with the fibrous connective structures, the associated vasculature or the adipocytes. In this case, cells obtained by fat wash could represent niches of stem cells easily detachable from fat tissue and/or the consequence of surgical procedure. In line with this idea, in a preliminary set of experiments, we isolate ADSCs simply by fat wash from adipose tissue excised by surgical resection (unpublished data). Finally, the possible trans-differentiation of mature adipocytes or committed preadipocytes, that remain still controversial, needs to be investigated in the future. Results obtained by the comparative analysis of cells isolated by a standardized commercial device based on the mechanical separation and cells isolated with simple and economic laboratory-assisted methods did not support the application of these category of high-priced systems even if the risk of contamination and the general safety of the process is higher using these type of devices. Moreover, mechanical manipulation of samples produces large amounts of oil residues and cellular debris that could affect the engraftment and the final surgical outcome. The number of cells isolated by collagenase digestion, considered the reference standard method, is significantly higher with yields ranging from 8 to 20-fold increase (data not shown) compared to isolation in the absence of enzymatic dissociating agents. However, according to the criteria indicated by the regulatory authorities to avoid the risk of toxins and xenobiotics contamination associated to the enzymes used to dissociate the adipose tissue, non-enzymatic isolation systems are required for clinical use. A recent study comparing research-grade collagenase with animal-free and xeno-free alternative products did not show any differences in cell yield, proliferation, surface marker phenotype or differentiation capacity^48^, but their current use may be limited by the cost. Moreover, even if the enzymatic dissociation of fat resulted in the best results in *in vitro* cell culture, there is not clinically relevant proof that it positively impacts long-term outcome *in vivo*. Data presented here, highlight the discussion about the value of ADSCs associated to the fluid portion of lipoaspirates, frequently discarded as a by-product of surgical procedure, and of the application of these cells alone or as cell-assisted lipotransfer in combination with fat tissue. ADSCs enriched transplants, combining fat grafting with ADSCs therapy, is still used primarily for research and tissue engineering because it is believed that more evidence-based medicine is needed to support its use. In mice, ADSCs-supplemented fat grafts had nrichment as it has been observed that autologous mesenchymal stem cell transplantation increases the thickness of the dermis, neovascularization in local skin, collagen neoformation^49^ and enhances skin graft survival in diabetic rats^50^. Moreover, the demonstration that ADSCs differentiation is not restricted to the mesenchymal origins, suggests the use of these cells in combination with epidermal cells suspension for skin repair. Up to now, the possibility to reprogram adult MSCs into melanocytes has been only documented in a restricted cell population named multilineage-differentiating stress-enduring (MUSE) cells, recently discovered in the bone marrow, adipose tissue, and dermis^51–53^. Here, immunoa longer survival than fat tissue alone^54^. Cell-assisted lipotransfer has rarely been used clinically in human patients to fill large soft tissue defects^55^, or for cosmetic breast augmentation^56^. We propose that, since ADSCs extracted from the liquid phase and by fat tissue wash could be combined and injected in a minimal volume (few microliter), the methods described here may be useful for the clinical applications of ADSCs therapy alone or in combination for fat minigrafts, a frequent practice in dermatology. In such cases, fat minigrafts are preferred due to minimal volume defect, with a consequent limitation in number of stem cells. MSCs populations have been shown to be present with lower frequency in many body areas including the skin. Extraction from the skin, the most accessible organ among the source of MSCs, unfortunately involves a complicated series of cell culture processes over several weeks. Therefore, to treat injuries, diseases, or cosmetic problems of the skin surgeons and dermatologists are taking advantage of regenerative cells found within adipose tissue. In this specific surgical field, the ideal technical improvement is to minimize graft volume and to maximize functional and aesthetic tissue restoration. We recently successfully used fat minigrafts in combination with autologous non-cultured epidermal cell suspension transplantation to correct skin scarring occurring following skin cancer resection^57^. This procedure, which produces better results compared to autologous full-thickness skin graft, could be improved in the future by non-enzymatic ADSCs estaining for melanogenic specific proteins suggests a homogeneous trans-differentiation into melanocyte phenotype at least *in vitro*. The evidence that ADSCs also act as a source for melanocytes suggests that this system could contribute not only to understanding human melanocyte biology, but also to therapeutic treatments of various pigment cell disorder, including vitiligo.

Studies dealing with the influence of age on ADSC yields and proliferation rates vary tremendously in their outcomes. Several studies have found a negative correlation between cell yield and donor age^12, 58, 59^. Other reports deny such a correlation and claim that the age of the donors does not influence the cell yield^60–62^. It is important to underline that all these evaluations are based on samples processed by collagenase digestion, that the enzymatic activity varies between batches, and that concentrations are usually given in weight per volume percent (*w/v*) resulting in irregularities between different isolations even when the same protocol is used. The present study, shows that no significant correlation exists between age and the general outcome of cell cultures evaluated in terms of cell viability, adhesion on plastic surface and cell proliferation when lipoaspirates are processed by enzyme-free minimal manipulation. This observation implies that the clinical use of the proposed methods do not depend on the age of the patient.

In conclusion, considering that adipocytes and ADSCs are both needed for tissue enlargement, but therapies for improving the quality of tissue may not need any adipocytes, we propose new types of processed adipose tissue (without adipocytes) to be used in the future as an alternative to fat grafting for nonvolumizing purposes, such as revitalization of stem cell-depleted tissue. This innovative approach completely overturns the initial idea of adipose tissue transplantation, according to which the liquid fractions of lipoaspirates constitute a by-product of the sampling phase giving new therapeutic opportunities in regenerative medicine.

## Materials and Methods

### Materials

Fetal bovine serum (FBS), penicillin-streptomycin mix, Trypsin-EDTA, and Phosphate Buffered Saline (PBS), Dulbecco’s modified Eagle’s medium DMEM were provided from EuroClone (Milan, Italy). Hanks balanced salt solution (HBSS), Collagenase A, 1-methyl-3-isobutylxanthine (IBMX), dexamethasone, indomethacin, beta-glycerol phosphate, ascorbic acid, insulin, Oil-Red O, Alizarin Red S, ammonium hydroxide, Triton-X100, Tween 20, 4′,6′-diamidino-2-phenylindole (DAPI), were provided from Sigma-Aldrich Srl, (Milan, Italy). BCIP-NBT substrate was purchased from Roche Diagnostics GmbH (Mannheim, Germany).

### Adipose tissue sampling

Specimens were obtained from patients treated with lipoaspirates transplants and enrolled by the Division of Plastic and Reconstructive Surgery, San Gallicano Dermatologic Institute, of the (IFO). The Declaration of Helsinki Principles was followed and patients gave written informed consent to collect samples of human material for research. Furthermore, the Institutional Research Ethics Committee (Istituti Regina Elena e San Gallicano) approved all research activities involving humans. All samples, harvested from the abdominal area, were waste materials collected as a by-product of surgery. The mean age of patients was 50 ± 12 years (n = 33; women n = 21 men n = 12) and ranged between 16 to 74 years.

### Surgical procedure to obtain lipoaspirates

Fat tissue was harvested under general anesthesia from the abdominal region with a 3 mm blunt cannula by standard sterile liposuction techniques as described by Coleman^36^ with infiltration of Kleine’s solution (30cc/100 cm^2^) using 20cc Luer-lock syringes.

### Adipose-derived stem cell isolation and purification

Discarded adipose tissue was collected during surgery, immediately transported to the laboratory and processed upon receipt. The protocols below describe in detail how the ADSCs were isolated in our laboratory. The entire isolation process, from the end of the harvest to the delivery of cells, was completed within 60–90 min.

i. Recovery from the fluid portions: a portion of the liquid fraction was immediately recovered during the surgical procedure and analyzed separately (intra-operative fast sedimentation). To additionally separate the liquid phase the harvested material collected in syringes (~8 mL) was processed using two different methods: a) by centrifugation according to the Coleman technique (3 min at 3000 rpm e.g. 1,811 *g*) and then left to decant for an additional 10 min; b) a sedimentation step (20 min). In both cases, cells were then collected from the fluid portion (2–3 mL) by centrifugation at 300 *g* for 5 minutes at room temperature and the pellets were suspended in red blood cell lysis buffer (160 mM NH_4_Cl) and incubated at 37 °C for 5 min. Cell suspension was filtered through a 70 μm cell strainer (BD Bioscences, Milan, Italy) and centrifuged at 300 *g* for 5 min. The upper phase containing fat tissue was further treated as reported below.
ii. The isolation kit (Faststem, Corios San Giuliano Milanese, Italy) is a sterile, single use, manual device consisting of a tissue collection container in which cells from adipose tissue are isolated mechanically in absence of enzymatic dissociating agents by massaging the outside of the bag and subsequently recovering of the material with a syringe. Recovered fat and fluid portions were separated by centrifugation of syringes at 300 *g* for 10 minutes. Harvested material collected in a syringe (~8 mL) was used to separate broken fat (upper phase) and the liquid fraction (lower phase). To concentrate the cells, the fluid fraction (~1–2 mL) was then centrifuged at 300 *g* for 5 min. The upper phase containing fat tissue was treated further as reported below.
iii. Fat wash (upper phase): 2–3 ml of adipose tissue (mechanically broken or not separated from aspirated fluid) were mixed with 3 volumes of PBS and incubated at 37 °C in a water bath for 30 minutes. The tubes were shaken vigorously every 5 minutes. Cells were separated from the adipocyte fraction by centrifugation at 300 *g* for 5 min at room temperature. The pellets were suspended in red blood cell lysis buffer (160 mM NH_4_Cl) and incubated at 37 °C for 5 min. Cell suspension was filtered through a 70 μm cell strainer (BD Bioscences) and centrifuged at 300 *g* for 5 min.
iv. Enzymatic digestion of fat (upper phase): the lipoaspirates (2–3 ml) were digested with an equal volume (2–3 mL) 0.075% collagenase A solution in HBSS at 37 °C in a water bath. The tubes were shaken vigorously every 5 min. The digestion was stopped after 30 minutes by adding 5 mL DMEM + 10% FBS and the digested sample was centrifuged at 300 *g* for 5 min at room temperature. Fat and oil were discarded and the pellets were suspended in red blood cells lysis buffer (160 mM NH_4_Cl) and incubated at 37 °C for 5 min. Cell suspension was filtered through a 70 μm cell strainer (BD Bioscences) and centrifuged at 300 *g* for 5 min.

In all the cases described above pellets containing the stromal vascular fraction (SVF) were seeded onto one T25 flask incubated at 37 °C, 5% CO_2_ in DMEM containing 40% FBS and antibiotics to select adherent cells. The remaining floating cells were aspirated off 48 hours later, and the flask was washed with DMEM to remove any debris. ADSCs were maintained in DMEM supplemented with 40% FBS in humidified at 5% CO_2_ 37 °C for 14 days. At this point, cells (live without trypan blue and dead with Trypan Blue) were counted in duplicate using TC20 automated cell counter (BioRad) and expanded for experiments in DMEM containing 20% FBS. Cell yields were normalized by dividing the cell number by the initial volume (in mL) of the lipoaspirate portion processed.

### Flow cytometric analysis and phenotypic characterization

At passage 2 cells were harvested by incubation in 0.5% trypsin, 0.2% ethylenediamine tetraacetic acid (EDTA) and examined by flow cytometric analysis (FACS) to evaluate the expression of stem cell-specific surface antigens (CD105, CD73, CD90, and CD44) and to exclude the expression of hematopoietic and endothelial cell populations (CD45, CD34, CD29, CD14, CD11b and HLA-DR). In addition, cells were immunostained with CD49d and CD54 antibodies. 3 × 10^5^ cells were incubated at RT for 30 min with specific antibodies diluted in PBS. After washing in PBS, cell suspensions (2 × 10^4^ cells per sample) were analyzed on a FACS Calibur instrument (BD) equipped with FlowJo software v8.0 by gating at 3% for each marker. All antibodies were purchased from BD Biosciences.

### Cell proliferation

Cells, at passages 2 and 6 were harvested using trypsin-EDTA and placed, in duplicate, in three different 24-well culture plates at a density of 4 × 10^3^ cells/cm^2^ and left to growth overnight (T0), 3 or 6 days before being harvested by incubation in trypsin-EDTA. Cell number and viability was measured by Trypan Blue exclusion assay using TC20 automated cell counter (BioRad). The increase in cell growth was calculated as a percentage of the number of cells at the indicated time points with respect to T0.

### Multilineage Cell Differentiation

i. Adipogenic: cells were plated in 12-well plates and grown until approximately 80% confluent, followed by adipogenic differentiation in DMEM containing 10% FBS, 0.5 mM isobutyl-methyl xanthine (IBMX), 1 µM dexamethasone, 10 µM insulin, and 200 µM indomethacin, replacing the medium every 2–3 days. The plates were maintained for 1 week until RNA extraction (6-well plate) and 2 weeks until lipid droplet formation was evaluated (12-well plate). For this purpose, fixed cells (in 4% paraformaldehyde for 10 min) were washed with 60% isopropanol and stained with Oil Red O to visualize lipid droplets. Cells were then washed with isopropanol and counterstained with hematoxylin. Light microscopy images were captured before semi-quantification. For quantification of lipid accumulation, the Oil Red O was extracted with isopropyl alcohol and the absorbance at 540 nm was measured against the blank solvent using a spectrophotometer. Each sample was differentiated and stained in duplicate. Undifferentiated cells were used as control which was normalized as 100%.
ii. Osteogenic: 100% confluent ADSCs were cultured with osteogenic differentiation media containing DMEM, 10% FBS, 0.1 µM dexamethasone, 10 mM β-glycerolphosphate, 50 µM ascorbate-2-phosphate, replacing the medium every 3 days. The plates were maintained for 1 week until RNA extraction and 2 weeks until fixing in 10% (v/v) formaldehyde (20 min) for mineralization evaluation and (1 min) for alkaline phosphatase (AP) activity assay. The cells were then stained using Alizarin Red S (40 mM; pH 4.1), which specifically stains calcium deposits. Plates were then washed four times with Milli-Q water. Stained monolayers were visualized by phase microscopy using an inverted microscope (Nikon). For quantification of staining, 800 µl 10% (v/v) acetic acid was added to each well, and the plate was incubated at room temperature for 30 min while shaking before transfering the sample to a 1.5 mL microcentrifuge tube and vortex. The extracts were heated to 85 °C for 10 min and then centrifuged at 13,000 rpm for 15 min. 500 µl of the supernatant were transfed to a new tube and neutralized adding 200 µl of 10% (v/v) ammonium hydroxide. Aliquots of the supernatant were read in triplicate at 405 nm in 96-well format plates. The results were expressed as percentages of the respective controls (non-differentiated cells), which were normalized as 100%. Following a permeabilization step with 0.05% Tween 20 in PBS, AP activity, was detected incubating cellular monolayer with BCIP/NBT as substrate, which stains cells blu-violet when AP is present, in the dark for 2 hours. Plates were then washed with PBS-Tween 20 (0.05%) and visualized by phase microscopy using an inverted microscope.
iii. Neurogenic: 80% confluent ADSCs were induced with Neurogenic Differentiation Medium (PromoCell, Heildeberg, Germany) changing the medium every third day for 5 days until RNA extraction (6-well plate) or 8 days until fix and stain for neuronal marker staining.
iv. Melanogenic: cells were directly plated in medium for the culture of human melanocytes (M254 supplemented with HMGS, Cascade Biologics, Incs. Portland, USA) the same day of cell isolation, since preliminary unpublished data demonstrated a higher rate of expression of melanogenic specific markers. Medium was replaced every 3 days.

In all cases cells cultured in DMEM 10% FBS were used as controls.

Images were recorded using an Axiovert 25 inverted microscope (Carl Zeiss, Oberkochen, Germany) and a Power Shot G5 digital camera (Canon, Inc., Tokyo, Japan).

### Semi-quantitative RT-PCR

Total RNA was extracted using Aurum Total mini kit (BioRad, Milan Italy). cDNA was synthesized from 1 μg of total RNA using a FirstAid kit (Fermentas, ThermoFisher Scientific, Waltham, MA, USA) and amplified in a reaction mixture containing iQSYBR Green Supermix (BioRad) and 25 pmol of forward and reverse primers using an iQ5 Light Cycler (BioRad). All samples were run in triplicate. The expression of sterol regulatory element-binding protein 1 (SREBP1), lipo protein lipase (LPL) and peroxisomal proliferator-activated receptor (PPARγ), fatty acid desaturase 2 (FADS2); bone sialophosphoprotein (BSP), runt-related transcription factor (RUNX) 2, cwcv and kazal-like domains proteoglycan (SPOCK1), osterix; sex determining region Y-box 2 (Sox-2), fascin, nestin, tubulinβIII; microphthalmia-associated protein (MITF), tyrosinase, and tyrosinase-related protein 2 (TRP2) transcripts were assessed by quantitative reverse transcription to determine the lineage-specific gene expression profiles. Amplification of the glyceraldehyde-3-phosphate dehydrogenase (GAPDH) transcript from each sample was included as internal control. Sequences of primers (intron spanning) can be found in Table S1. For each gene, the assessment of quality was performed by examining PCR melt curves after qRT-PCR to ensure product specificity.

### Immunofluorescence analysis

Cells were fixed with 4% paraformaldehyde for 20 min at room temperature followed by 0.1% Triton X-100 to allow cell permeabilization. Cells were then incubated with the following primary antibodies: anti-PPARγ, anti-SREBP1, anti-RUNX2, anti-Mitf, anti-Tyrosinase, anti-TRP2 (Santa Cruz Biotechnology, USA), anti-Sox2, anti-Nestin antibody (Merck Millipore, Germany), for 1 hour. Primary antibodies were visualized using anti-rabbit IgG, anti-goat or anti-mouse IgG Alexa Fluor 488 (BD Bioscences). Nuclei were visualized with DAPI. Fluorescence signals were recorded using a CCD camera (Zeiss, Oberkochen, Germany).

### Statistical analysis

In the figures, single experiment and results are representative of several experiments we performed with at least five adipose-derived stem cells isolated from different donors. Quantitative data were obtained in duplicates or triplicates and reported as mean ± standard deviation (SD). The data were statistically analyzed using Student *t*-test. A p value of less than 0.05 was considered significant.

## Electronic supplementary material

Supplementary Information

## References

[1] van Harmelen V, Skurk T, Hauner H (2005). Primary culture and differentiation of human adipocyte precursor cells. Methods Mol Med.

[2] Zuk PA (2001). Multileneage cells from human adipose tissue: implications for cell-base therapies. Tissue Eng.

[3] Zuk PA (2002). Human adipose tissue is a source of multipotent stem cells. Mol Biol Cell.

[4] da Silva Meirelles L, Caplan AI, Nardi NB (2008). In search of the *in vivo* identity of mesenchymal stem cells. Stem Cells.

[5] Qin Y, Zhou C, Wang N, Gao WQ (2015). Conversion of adipose tissue-derived mesenchymal stem cells to neural stem cell-like cells by a single transcription factor, Sox-2. Cell Reprogram.

[6] Wakao S, Matsuse D, Dezawa M (2014). Mesenchymal stem cells as a source of Schwann cells: their anticipated use in peripheral nerve regeneration. Cells Tissue Organs.

[7] Khorsandi L, Khodadadi A, Nejad-Dehbashi F, Saremy S (2015). Three-dimensional differentiation of adipose-derived mesenchymal stem cells into insulin-producing cells. Cell Tissue Res.

[8] Yin L (2015). Adipose tissue-derived m,esenchymal stem cells differentiated into hepatocyte-like cells *in vivo* and *in vitro*. Mol Med Rep.

[9] Daley GQ (2012). Cellular alchemy and the golden age of reprogramming. Cell.

[10] Engle SJ, Puppala D (2013). Integrating human pluripotent stem cells into drug development. Cell Stem Cell.

[11] Tabit CJ, Slack GC, Fan K, Wan DC, Bradley JP (2012). Fat grafting versus adipose-derived stem cell therapy: distinguishing indications, techniques, and outcomes. Aesth Plast Surg.

[12] Aust L (2004). Yield of human adipose-derived adult stem cells from liposuction aspirates. Cytotherapy.

[13] El-Badawy A (2016). Adipose stem cells display higher regenerative capacity and more adaptable electro-kinetic properties compared to bone marrow-derived mesenchymal stromal cells. Sci Rep.

[14] Yoshimura K (2006). Characterization of freshly isolated and cultured cells derived from the fatty and fluid portions of liposuction aspirates. J Cell Physiol.

[15] Gimble JM, Bunnell BA, Chiu ES, Guilak F (2011). Concise review: Adipose-derived stromal vascular fraction cells and stem cells: let’s not get lost in translation. Stem Cells.

[16] Konczalik W, Siemionow M (2014). Experimental and clinical methods used for fat volume maintenance after autologous fat grafting. Ann Plast Surg.

[17] Forcales SV (2015). Potential of adipose-derived stem cells in muscular regenerative therapies. Front Aging Neurosci.

[18] Zimmerlin L (2011). Regenerative therapy and cancer: *in vitro* and *in vivo* studies of the interaction between adipose-derived stem cells and breast cancer cells from clinical isolates. Tissue Eng Part A.

[19] Coleman SR, Katzel EB (2015). Fat grafting for facial filling and regeneration. Clin Plast Surg.

[20] Magalon G (2015). Regenerative approach to scleroderma with fat grafting. Clin Plast Surg.

[21] Zimmerlin L, Park TS, Zambidis ET, Donnenberg VS, Donnenberg AD (2013). Mesenchymal stem cell secretome and regenerative therapy after cancer. Biochimie.

[22] Klinger M (2015). Regenerative approach to scars, ulcers and related problems with fat grafting. Clin Plast Surg.

[23] Simonacci F, Bertozzi N, Grieco MP, Grignaffini E, Raposio E (2016). Autologous fat transplantation for breast reconstruction: A literature review. Ann Med Surg.

[24] Pu LL (2012). Toward more rationalized approach to autologous fat grafting. J Plast Aesthet Surg.

[25] Kuno S, Yoshimura K (2015). Condensation of tissue and stem cells for fat grafting. Clin Plast Surg.

[26] Kokai LE, Marra K, Rubin JP (2014). Adipose stem cells: biology and clinical applications for tissue repair and regeneration. Transl Res.

[27] Matsumoto D (2006). Cell-assisted lipotransfer: supportive use of human adipose-derived cells for soft tissue augmentation with lipoinjection. Tissue Eng.

[28] Kolle SF (2013). Enrichment of autologous fat graft with *ex-vivo* expanded adipose tissue-derived stem cells for graft survival: a randomized placebo-controlled trial. Lancet.

[29] Carvalho PP, Gimble JM, Dias IR, Gomes ME, Reis RL (2013). Xenofree enzymatic products for the isolation of human adipose-derived stromal/stem cells. Tissue Eng Part C Meth.

[30] Kirkpatrick CJ, Melzner I, Göller T (1985). Comparative effects of trypsin, collagenase and mechanical harvesting on cell membrane lipids studied in monolayer-cultured endothelial cells and green monkey kidney cell line. Biochim Biophys Acta.

[31] Jahr H, Hering B, Federlin K, Bretzel RG (1995). Activation of human complement by collagenase and ficoll. Exp. Clin. Endocrinol. Diabetes.

[32] Pilgaard L, Lund P, Rasmussen JG, Fink T, Zachar V (2008). Comparative analysis of highly defined proteases for the isolation of adipose tissue-derived stem cells. Regen Med.

[33] Bianchi F (2012). A new nonenzymatic method and device to obtain a fat tissue derivate highly enriched in pericyte-like elements by mild mechanical forces from human lipoaspirates. Cell Transplant.

[34] Reinhardt M, Bader A, Giri S (2015). Devices for stem cell isolation and delivery: current need for drug discovery and cell therapy. Expert Rev Med Devices.

[35] Shah FS, Wu X, Dietrich M, Rood J, Gimble JM (2013). A non-enzymatic method for isolating human adipose tissue-derived stromal stem cells. Cytotherapy.

[36] Coleman SR (2006). Structural fat grafting: more than a permanent filler. Plast Reconconstr Surg.

[37] Dominici M (2006). Minimal criteria for defining multipotent mesenchymal stromal cells. The International Society for Cellular Therapy position statement. Cytotherapy.

[38] Krampera M, Galipeau J, Shi Y, Tarte K, Sensebe L (2013). Immunological characterization of mesenchymal stromal cells- The international Society for Cellular Therapy (ISCT). Cytotherapy.

[39] Cawthorn WP, Scheller EL, MacDougald OA (2012). Adipose tissue stem cells meet preadipocyte commitment: going back to the future. J Lipid Res.

[40] De Ugarte DA (2003). Differential expression of stem cell mobilization-associated molecules on multi-lineage cells from adipose tissue and bone marrow. Immunol Letters.

[41] Bartosh TJ (2010). Aggregation of human mesenchymal stromal cells (MSCs) into 3D spheroids enhances their antiinflammatory properties. Proc Nat. Acad Sci USA.

[42] Bartosh TJ, Ylöstalo JH, Bazhanov N, Kuhlman J, Prockop DJ (2013). Dynamic compaction of human mesenchymal stem/precursor cells into spheres self-activates caspase-dependent IL1 signaling to enhance secretion of modulators of inflammation and immunity (PGE2, TSG6, and STC1). Stem Cells.

[43] Bazhanov N (2016). Intraperitonealy infused human mesenchymal stem cells from aggregates with mouse immne cells and attach to peritoneal organs. Stem Cell Res.

[44] Li L, Bhatia R (2011). Stem cell quiescence. Clin Cancer Res.

[45] Francis MP, Sachs PC, Elmore LW, Holt SE (2010). Isolating adipose-derived mesenchymal stem cells from lipoaspirates blood and saline fraction. Organogenesis.

[46] Yoshimura K, Suga H, Eto H (2009). Adipose-derived stem/progenitors cells: roles in adipose tissue remodeling and potential use for soft tissue augmentation. Regen Med.

[47] Crisan M (2008). A perivascular origin for mesenchymal stem cells in multiple human organs. Cell Stem Cell.

[48] Carvalho PP, Gimble JM, Dias IR, Gomes ME, Reis RL (2013). Xenofree enxymatic products for the isolation of human adipose-derived stromal/stem cells. Tissue Eng Part C Methods.

[49] Mojallal A (2009). Improvement of skin quality after fat grafting: clinical observation and an animal study. Plast Reconstr Surg.

[50] Sheng L (2011). Transplantation of adipose stromal cells promotes neovascularization of random skin flaps. J Exp Med.

[51] Tsuchiyama K (2013). Functinal melanocytes are readily reprogrammable from multilineage-differentiating stress-enduring (muse) cells, distinct stem cells in human fibroblasts. J Invest Dermatol.

[52] Ogura F (2014). Human adipose tissue possesses a unique population of pluripotent stem cells with nontumorigenic and low telomerase activities: potential implications in regenerative medicine. Stem Cells Dev.

[53] Wakao S, Akashi H, Kushida Y, Dezawa M (2014). Muse cells, newly found non-tumorigenic pluripotent stem cells, reside in human mesenchymal tissue. Pathol Int.

[54] Fu BC, Gao JH, Lu F, Li J (2010). Experimental study of the effect of adipose stromal vascular fraction cells on the survival rate of fat transplantation. Zhonghua Zheng Xing Wai Ke Za Zhi.

[55] Yoshimura K (2008). Cell-assisted lipotransfer for facial lipotrophy: efficacy of clinical use of adipose-derived stem cells. Dematol Surg.

[56] Yoshimura K (2008). Cell-assisted lipotransfer for cosmetic breast augmentation: supportive use of adipose-derived stem/stromal cells. Aesthetic Plast Surg.

[57] Migliano, E., Bellei, B., Govoni, F.A., Bucher, S. & Picardo, M. Fat and epidermal cell suspension grafting: a new advanced one-step skin regeneration surgical techniques. *J exp Clin Cancer Res* **24**, 1186/1756-9966-33-23. 10.1186/1756-9966-33-23 PMC3975962 24565036

[58] Choudhery MS, Badowski M, Muise A, Pierce J, Harris DT (2014). Donor age negatively impacts adipose tissue-derived mesenchymal stem cell expansion and differentiation. J Trans Med.

[59] Dos-Anjos Vilaboa S, Navarro-Palou M, Llull R (2014). Age influence on stromal vascular fraction cell yield obtained from human lipoaspirates. Cytotherapy.

[60] Buschmann J (2013). Yield of purification rate of adipose-derived stromal cells as a function of age, body mass index and harvest site-increasing the yield by use of adherent and supernatant fractions?. Cytotherapy.

[61] Faustini M (2010). Nonexpanded mesenchymal stem cells for regenerative medicine: yield in stromal vascular fraction from adipose tissues. Tissue Eng.

[62] Mojallal A (2011). Influence of age and body mass index on the yield and proliferation capacity of adipose-derived stem cells. Aesth Plast Surg.

