# Supplementary figures and images for: Maximizing non-enzymatic methods for harvesting adipose-derived stem from lipoaspirate: technical considerations and clinical implications for regenerative surgery
Source: Sci Rep. 2017 Aug 30;7:10015. doi: 10.1038/s41598-017-10710-6 (PMC5577104; doi:10.1038/s41598-017-10710-6)

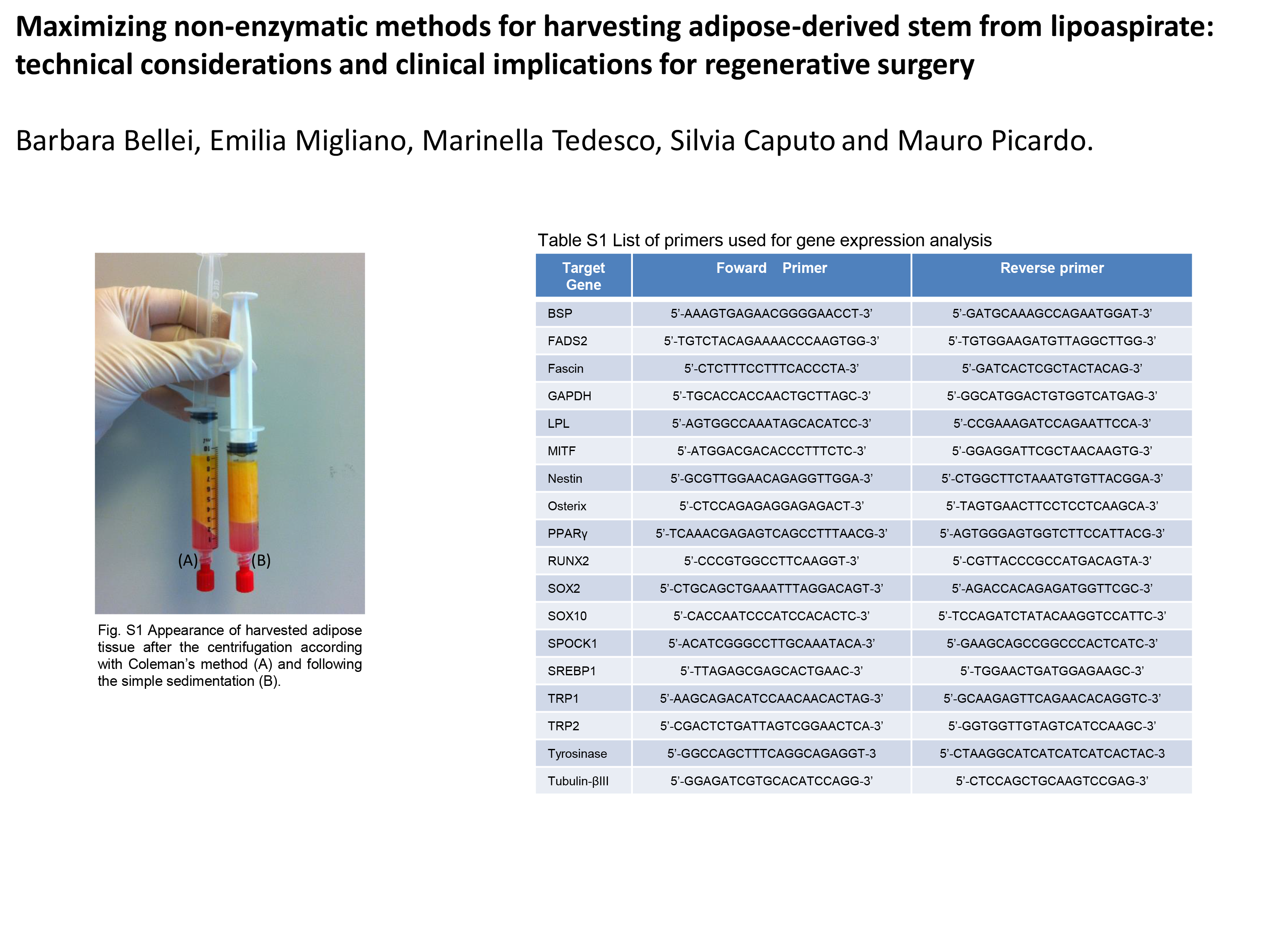

Supplement: Supplementary file 1 — Supplementary Information [file 41598_2017_10710_MOESM1_ESM.tif]
